# The Influence of Emotional Material on Encoding and Retrieving Intentions: An ERP Study in Younger and Older Adults

**DOI:** 10.3389/fpsyg.2018.00114

**Published:** 2018-02-16

**Authors:** Alexandra Hering, Matthias Kliegel, Patrizia S. Bisiacchi, Giorgia Cona

**Affiliations:** ^1^Faculty of Psychology and Educational Sciences, University of Geneva, Geneva, Switzerland; ^2^Center for the Interdisciplinary Study of Gerontology and Vulnerability, University of Geneva, Geneva, Switzerland; ^3^Swiss National Center of Competences in Research LIVES – Overcoming Vulnerability: Life Course Perspectives, Geneva, Switzerland; ^4^Department of General Psychology, University of Padua, Padua, Italy; ^5^Padua Neuroscience Center, Padua, Italy

**Keywords:** prospective memory, aging, partial least square analysis, emotion, EEG

## Abstract

Prospective memory is a cognitive process that comprises the encoding and maintenance of an intention until the appropriate moment of its retrieval. It is of highly relevance for an independent everyday life, especially in older adults; however, there is ample evidence that prospective memory declines with increasing age. Because most studies have used neutral stimuli, it is still an open question how emotional factors influence age-related differences in prospective remembering. The aim of the study was to investigate the influence of emotional material on prospective memory encoding, monitoring, maintaining, and retrieval in younger and older adults using behavioral and electrophysiological measures. We tested 24 younger adults (*M* = 26.4 years) and 20 older adults (*M* = 68.1 years) using a picture one-back task as ongoing activity with an embedded prospective memory instruction. The experimental task consisted of three sessions. In each session, participants had to encode series of images that represented the prospective memory cues for the consecutive block. The images were either of pleasant, unpleasant, or neutral valence. The pictures used in the ongoing task were likewise of pleasant, unpleasant, or neutral valence. Event-related potentials (ERPs) were recorded to assess the neural correlates of intention encoding, maintenance, and self-initiated retrieval. We did not find age differences between younger and older adults on the behavioral level. However, the ERP results revealed an interesting pattern that suggested for both age groups elevated attentional processing of emotional cues during encoding indicated by an elevated LPP for the emotional cues. Additionally, younger adults showed increased activity for unpleasant cues. During the maintenance phase, both age groups engaged in strategic monitoring especially for pleasant cues, which led to enhanced sustained positivity. During retrieval, older adults showed increased activity of ERP components related to cue detection and retrieval mainly for pleasant cues indicating enhanced relevance for those cues. In conclusion, emotional material may influence prospective remembering in older adults differently than in younger adults by supporting a mixture of top-down and bottom-up controlled processing. The results demonstrated a negativity bias in younger adults and a positivity bias in older adults.

## Introduction

Remembering to buy bread on the way home and remembering to do the taxes on the weekend are typical examples of prospective memory tasks in everyday life. Prospective memory describes the ability to remember and execute delayed intentions in the future (Kliegel et al., 2008a). Although remembering to buy bread on the way home is a relatively easy task, it still might be forgotten when we are stressed by work or excited to go to birthday drinks after work. Based on everyday life experience, it is comprehensible that emotions cannot only interact but also interfere with planned intentions and goals. Surprisingly, studies on emotional influences on prospective memory emerged only recently leaving still lots of open questions, especially with respect to the influence of emotions on age differences in prospective memory. The present study investigated how emotional material modulates the encoding, maintaining, and retrieving of intentions in younger and older adults.

### Prospective Memory and Aging

Remembering a delayed intention consists of four different processes with each relying on different cognitive functions (Kliegel et al., 2011). In a first step, the intention has to be encoded and planned for later execution; planning abilities are implied in this phase (e.g., Hering et al., 2014). In the following, the intention has to be maintained in memory while people are engaged in background activities – so-called ongoing tasks – until the appropriate moment for intention retrieval. The retrieval can be cued either by a certain moment in time labeled time-based prospective memory (e.g., remembering to take the pizza out of the oven after 20 min) or by a specific event labeled event-based prospective memory (e.g., remembering to withdraw money at the next cash maschine; Einstein and McDaniel, 1990). The retrieval and execution of intentions has mainly been associated with controlled cognitive functions such as task switching and inhibition (Schnitzspahn et al., 2013; Zuber et al., 2016). We have to interrupt the ongoing task and switch to the prospective memory task for its execution before returning to the ongoing activity. Conceptually, Kliegel et al. (2011) argued that a mismatch between the task demands in each of the four phases of prospective remembering and the available cognitive resources required for that specific phase determines age-related performance differences.

Several meta-analyses showed a general decline of prospective memory with increasing age across adulthood in laboratory prospective memory tasks (Henry et al., 2004; Kliegel et al., 2008b; Cona et al., 2012a; Ihle et al., 2013). Moreover, Ihle et al. (2013) showed that prospective memory tasks that rely on strategic monitoring are especially impaired in older adults. Strategic monitoring describes the allocation of attentional resources to detect the prospective memory cue within the ongoing task (McDaniel and Einstein, 2007a). In older adults, prospective memory decrements are accompanied by decrements in attentional and cognitive control capacities (e.g., Park and Reuter-Lorenz, 2009), resulting in decreases in strategic monitoring and reduced prospective memory performance (e.g., Smith and Bayen, 2006).

However, McDaniel and Einstein (2000) suggested an alternative route of prospective memory retrieval besides the top-down strategic monitoring. They argue that the retrieval of an intention can be triggered by the cue in a spontaneous and reflexive fashion due to processes of familiarity, distinctiveness, or discrepancy (McDaniel and Einstein, 2007b). For example, McDaniel and Einstein (2007b) hypothesized that if the prospective memory cue is very salient or distinct within the ongoing task, it is likely to provoke a spontaneous retrieval of the associated action. Similarly, very familiar cues and cues that are highly discrepant of the ongoing task will result in spontaneous retrieval because these cues capture attention more easily.

Moreover, spontaneous retrieval is based on bottom-up processing (Bugg et al., 2013) and thus, it should be spared of decrements in older adults (Cherry et al., 2001; Cohen et al., 2003). For example, Cohen et al. (2003) used perceptually distinct cues and showed benefits on prospective memory performance in older adults. Importantly for the present purposes, we argue that using emotional material can also increase the distinctiveness of the prospective memory cue and eliminate age differences. This idea was first tested by Altgassen et al. (2010) who compared emotional prospective memory cues with neutral cues in younger and older adults and showed that older adults performed comparably to younger adults with both pleasant and unpleasant cues, but they performed worse than younger adults with neutral prospective memory cues indicating that emotional valence increases the distinctiveness of prospective memory cues. However, the emerging literature of emotional influences on prospective memory and in particular on age differences in prospective memory demonstrates some contradictory findings, especially regarding the underlying mechanisms.

### The Role of Emotions on Age-Related Differences in Prospective Memory

So far, only a handful of studies have addressed the role of emotional material on prospective memory in older adults (Altgassen et al., 2010; Rendell et al., 2011; Schnitzspahn et al., 2012; Ballhausen et al., 2015; May et al., 2015). Interestingly, the studies showed diverging results regarding the beneficial or disadvantageous influence on prospective memory performance, whereas the study by Ballhausen et al. (2015) found worse prospective memory retrieval for pleasant and unpleasant prospective memory cues compared to neutral cues; Altgassen et al. (2010), Rendell et al. (2011), and Schnitzspahn et al. (2012) showed that older adults improved their prospective remembering with emotional material.

More precisely, in the study by Ballhausen et al. (2015), participants encoded a semantic category (e.g., animals) as prospective memory cues along with emotional examples to manipulate the valence of encoding; however, the later actual cues within the task were of neutral valence (Experiment 2). The results showed that older adults performed worse with the emotional prospective memory cue examples than with neutral ones. The authors conducted another experiment, where they manipulated the valence of the actual prospective memory cue within the task (Experiment 1). Here, older adults showed no differences between pleasant, neutral, and unpleasant prospective memory cues at retrieval. Overall, the study suggested that emotional material might not always enhance prospective memory performance in older adults. Furthermore, emotional material seemed to be more relevant at the encoding phase than the retrieval phase.

The studies showing a beneficial effect of emotional material, however, vary in the magnitude of the effect. In the first study by Altgassen et al. (2010), age differences between younger and older adults were eliminated in prospective memory performance for pleasant and unpleasant prospective memory cues compared to neutral cues. The study by Schnitzspahn et al. (2012) only found that age differences between younger and older adults were attenuated for emotional prospective memory cues compared to neutral ones. Finally, Rendell et al. (2011) did only find an emotional enhancement effect for pleasant material in older adults.

However, when comparing these three studies to the findings reported by Ballhausen et al. (2015), differences in task design between the studies suggest already possible underlying mechanisms regarding the influence of emotional material on prospective memory. Ballhausen et al. (2015) manipulated the valence of the prospective memory cue either at encoding or at retrieval but not in both phases, whereas the other studies used emotional prospective memory cues at encoding and retrieval. This leads to the assumption that especially the encoding of emotional material is relevant for the modulating effects.

Taken together, the empirical evidence on the influence of emotional material on age effects in prospective memory indicates a beneficial effect of emotional material in older adults. It is assumed that the emotional material enhances the salience of the prospective memory cues, and thus supports the detection of the prospective memory cues within the ongoing task (Altgassen et al., 2010; Schnitzspahn et al., 2012). Furthermore, May et al. (2015) argued that the beneficial effect of emotional material does not lead to more strategic monitoring for the cue but rather supports cue detection by the more salient emotional cues *per se* and thus enhancing spontaneous retrieval. Support for this conclusion comes from a study investigating emotional influences on prospective memory in younger adults only (May et al., 2012). The authors found an advantage in prospective memory performance for pleasant and unpleasant prospective memory cues compared to neutral cues, but they did not find indication for elevated monitoring. To the contrary, the authors even reported less monitoring in the emotional conditions compared to the neutral one. However, the heightened effect of emotional material is not always found. Similar to Ballhausen et al. (2015), Clark-Foos et al. (2009) found a deteriorating influence of emotional material on prospective memory performance in younger adults. The authors argue that emotional material might increase task-irrelevant thinking and has a distracting influence toward cue detection.

In sum, although the majority of studies suggested a beneficial influence of emotional material, there is also contradictory evidence in younger and older adults (e.g., Ballhausen et al., 2015). Attempts to identify the underlying mechanisms focused on the role of emotional material either in enhancing the cue saliency and fostering spontaneous retrieval or in boosting the relevance of the cues and fostering strategic monitoring. So far, the evidence supports rather the first assumption (e.g., Altgassen et al., 2010; May et al., 2012; but see Cona et al., 2015a for more monitoring with emotional material). However, the debate of possible underlying mechanisms is mainly led on the behavioral level, which clouds the identification of relevant processes. One study addressed the issue by including electrophysiological measures from the electroencephalogram (EEG) to investigate the neural correlates of emotional cue effects in younger adults (Cona et al., 2015a). The EEG shows high temporal resolution that allows for a precise investigation of underlying neural processes with respect to the presentation of stimuli and to responses.

### Prospective Memory, Emotion, and EEG

The study by Cona et al. (2015a) examined event-related potentials (ERPs) for the different phases of prospective remembering and how these phases were modulated by emotional material. Participants had to encode either pleasant, neutral, or unpleasant prospective memory cues. For the ongoing task, participants worked on a picture one-back task that consisted of pleasant, neutral, and unpleasant ongoing task trials. The design allowed investigating ERPs at encoding, maintaining, and retrieving intentions by crossing the three levels of emotional valence in both tasks.

For the encoding phase, the authors found elevated activity for the late positive potential (LPP) for emotional compared to neutral prospective memory cues. The LPP is a centro-parietal sustained positivity that starts around 300 ms after stimulus onset and can last for 1000–2000 ms and that is increased for emotional material. It is assumed that the LPP reflects sustained attention toward the emotional material (Hajcak et al., 2011). Respectively, the elevated LPP in the study by Cona et al. (2015a) indicated that more attentional resources were recruited for encoding of emotional (pleasant and unpleasant) intentions. During the maintenance phase, the authors found sustained activity in the LPP mainly for pleasant ongoing task trials in blocks where participants had to remember pleasant prospective memory cues. The finding suggests a rather specific allocation of monitoring resources to the more relevant ongoing task trials (pleasant ongoing task trials), as the prospective memory cues that had to been detected were pleasant as well.

A similar effect was expressed on the behavioral level. The authors found increased reaction times for the ongoing task stimuli that had the same valence as the prospective memory cue. This effect is labeled stimulus specific interference effect (SSIE; Cohen et al., 2012) and suggests increased strategic monitoring in situations where the task material for prospective memory cues and the ongoing task overlap and thus increase interference between the two task demands. Cohen et al. (2012) could show that the SSIE can be explained theoretically by the two processes model of strategic monitoring (Guynn, 2003). The model suggests that strategic monitoring for the prospective memory cue consists of two processes, (1) being in a retrieval mode and (2) checking for the target. The retrieval mode describes a general preparedness for the prospective memory cue to arrive. In other words, attentional resources are allocated to monitor for the prospective memory cue. Target checking describes the flexible comparison of the stimulus in the environment with the representation of the prospective memory (target) cue. When there is a high match between these two, monitoring increases resulting in higher reaction times (Cohen et al., 2012). Accordingly, if the emotional valences between prospective memory cues and ongoing task stimuli match (e.g., both being pleasant pictures), reaction times should be higher and monitoring should increase as it was shown by Cona et al. (2015a) for pleasant material.

For the retrieval phase of prospective memory, there are two component complexes discussed in the literature [for a review, see West (2011)]. The two components refer to the cue detection and the retrieval of the intention. For cue detection, West et al. (2001) identified a negativity – the N300 – at occipital sites around 300–500 ms after stimulus onset that distinguishes between prospective memory cues and ongoing task trials. Along with the N300, there is a frontal positivity that is also associated with cue detection. The second component is the parietal positivity that is associated with the retrieval of the intention. The parietal positivity occurs at parietal electrodes around 400–1200 ms after stimulus onset and consists of three subcomponents (West, 2011). The three subcomponents are the P3b that is linked to task-relevant evaluation of the prospective memory cues (e.g., Hering et al., 2016); the old–new effect that is linked to the retrieval from memory (e.g., West and Ross-Munroe, 2002); and the prospective positivity that reflects the switching between the ongoing task and the prospective memory task (e.g., Bisiacchi et al., 2009). Cona et al. (2015a) found in their study that the emotional material modulated especially the frontal positivity and the parietal positivity. The greater frontal positivity for emotional material seemed to reflect more automatic, bottom-up processing to detect the prospective memory cue (i.e., FN400). The elevated parietal positivity, which was the result of the P3b and the parietal old–new effect, suggested an engagement of top-down strategic resources to retrieve the intention from memory. Based on this pattern of modulations, the results by Cona et al. (2015a) seem to indicate that emotional material not only triggers an automatic, bottom-up capture of attention but also boosts a greater allocation of top-down strategic processes.

However, it remains an open question if that would also hold true for older adults. Older adults show usually performance decreases in tasks that need strategic monitoring, whereas tasks that rely on spontaneous retrieval seemed to be spared of age-related performance decreases. They also show rather attenuated activity for the N300 and the parietal positivity compared to younger adults (e.g., West and Covell, 2001; West and Bowry, 2005). Similarly, it has been found that older adults show smaller LPPs (e.g., Wood and Kisley, 2006).

### The Present Study

The present study followed up on that previous study with the main objective of investigating the influence of emotional material on prospective memory in younger and older adults on distinct phases composing prospective remembering using ERPs. The study aimed to clarify the contradictory findings regarding emotional prospective memory tasks in older adults (Altgassen et al., 2010; Ballhausen et al., 2015) by investigating the underlying mechanisms. Therefore, we replicated the study by Cona et al. (2015a) and applied the paradigm in a group of younger and older adults.

Following the previous literature, we expected that older adults should perform worse than younger adults for neutral prospective memory cues reflecting the general (non-emotional) decline in prospective memory performance. For pleasant and unpleasant cues, age differences should be smaller or even eliminated, if older adults benefit from the emotional saliency of the cues.

On the neural level, we expected attenuated activity in older adults compared to younger adults for the LPP at encoding and retrieval and for the prospective memory-related ERPs. Furthermore, older adults tend to show a positivity bias that might be detectable in elevated activity for pleasant cues compared to neutral and unpleasant cues (Mather and Carstensen, 2005). The positivity bias (or positivity effect) in older age is a motivational cognitive processing style that is grounded in the socioemotional selectivity theory (Carstensen, 2006). This theory suggests that due to the perceived limited time in older adults, there is a shift of goals from future-oriented goals such as seeking new information toward more present-oriented goals such as emotional regulation to increase well-being. The positivity bias describes the preference for pleasant or positive information in attention and memory tasks in older adults (see Reed et al., 2014 for meta-analytical evidence).

The neural correlates for the maintenance phase might elucidate the role of emotional material on prospective memory in older adults. If emotional material boosts the attentional processing of the cues, it should also increase monitoring processes. Thus, we would expect smaller monitoring ERP activity in older adults compared to younger adults due to decrements in attentional capacities. However, if emotional material would foster the distinctiveness of prospective memory cues, following McDaniel and Einstein (2000), older adults would rather rely on spontaneous retrieval and should not show monitoring activity during the maintenance phase.

## Materials and Methods

### Participants

Forty-four participants took part in the study. Among them, 24 participants belonged to the younger adults’ group (*M* = 26.42 years; range = 21–52 years; 12 males) and 20 participants belonged to the older adults’ group (*M* = 68.05 years; range = 60–80 years; 8 males). The younger adults were recruited from the University of Geneva and participated for course credits. The older adults were recruited at local senior associations and libraries and received 20 CHF in reimbursement for their time. To assess general cognitive abilities, we administered the matrices’ subtest and the vocabulary subtest from the Wechsler Adult Intelligence battery (WAIS 4; Wechsler, 2011). The two age groups did not differ regarding fluid intelligence assessed with the matrices subtest [*t*(42) = 1.846; *p* = 0.072; *M_young_* = 20.63; *SD_young_* = 3.51; *M_old_* = 18.65; *SD_old_* = 3.51] and verbal abilities assessed with the vocabulary subtest [*t*(42) = 1.313; *p* = 0.196; *M_young_* = 43.83; *SD_young_* = 6.28; *M_old_* = 46.05; *SD_old_* = 4.58]. All participants reported normal or corrected to normal vision and hearing, no history of neurological or major psychiatric diseases, and were not currently taking any psychoactive medication. All participants reported to be right-handed and were fluent in French or have spoken French for more than 5 years. Furthermore, we screened all older adults for their general cognitive status with the modified version of the French Telephone Interview for the Cognitive Status (F-TICS-m; Vercambre et al., 2010) and included only participants that scored higher than a value of 26 (*M_old_* = 36.50; *SD_old_* = 3.30). Given the study material, participants with specific phobias (e.g., blood phobia and snake phobia) were excluded. The study was approved by the ethic commission of the faculty of psychology and educational sciences of the University of Geneva. All participants signed an informed consent prior to the testing.

### Material

We used a prospective memory paradigm that was previously published by Cona et al. (2015a). The task consisted of emotional stimulus material from the International Affective Picture System (Lang et al., 2005); 228 pictures were selected for the task among them 76 pleasant, 76 unpleasant, and 76 neutral pictures. The selected pictures varied in their normative valence ratings to create the three different emotional conditions (pleasant pictures: *M* = 7.3; *SD* = 0.6; unpleasant pictures: *M* = 2.8; *SD* = 0.6; and neutral pictures: *M* = 5.0; *SD* = 0.2). Regarding the arousal of the three conditions, pleasant and unpleasant pictures did not differ from each other (pleasant pictures: *M* = 5.1; *SD* = 0.6; unpleasant pictures: *M* = 5.2; *SD* = 0.4; *p* > 0.05), but showed higher arousal ratings than neutral pictures (neutral pictures: *M* = 3.3; *SD* = 0.6; all *p* < 0.05). Pictures were chosen from four categories: persons, animals, landscape, and objects and represented a wide range of semantic categories to avoid biases for specific pictures. For more details on the picture selection, see Cona et al. (2015a). For each valence condition, 25 pictures were chosen that served as prospective memory cues and the remaining pictures were used as ongoing task material. Prospective memory cues and ongoing task stimuli were matched regarding valence and arousal ratings. The pictures were presented in the center of the computer screen in front of a black background.

The prospective memory task consisted of a one-back ongoing task with an embedded prospective memory instruction. For the ongoing task, participants had to judge if the presented picture on the screen was the same or a different picture as the previous one by pressing the corresponding keys. Participants had to press with the index and middle fingers of their right hand on the keys “G” or “H” of a French-Swiss keyboard for the same/different judgment. The response-key mapping for the same/different keys was counterbalanced across participants. On each trial, the picture appeared for 2000 ms or until a response was made, followed by a black screen with a fixation cross that pseudo-randomly lasted for 1200, 1400, or 1600 ms. The pictures were of pleasant, neutral, and unpleasant valence. In total, 24% of the ongoing task stimuli were one-back hits.

For the prospective memory instruction, participants had to press the “A” key with their left index finger after answering the ongoing task when seeing a previously encoded prospective memory cue. Before each block, participants had to encode five different pictures of the same valence with each picture presented for 2000 ms followed by a black screen for 1000 ms. Prospective memory cues were never one-back hits.

### Procedure

After signing the informed consent, the testing session started with the assessment of the general cognitive abilities and participants performed the matrices subtest and the vocabulary test. Afterward, the electrodes were applied on the head and in the face of the participants. The preparation for the EEG recording took approximately 15 min. The participants were installed in a noise shielded cabin in front of a computer, where the prospective memory task was administered.

The task instructions were displayed on the screen and participants were asked to explain all instructions in their own words to ensure understanding. The task started with a practice block to familiarize participants with the ongoing task (39 trials with 13 trials per valence). If they performed at least 85% correct, they could continue with the next block; otherwise, the instructions were explained again and participants could repeat the practice block. Following the practice, participants performed a baseline block for the ongoing task consisting of 198 trials.

Afterward, participants worked on three prospective memory sessions that represented the three valence conditions pleasant, neutral, and unpleasant referring to the respective valence of the prospective memory cues. The three sessions were counterbalanced across participants. The valence of the prospective memory cues in each session defined the valence condition (pleasant, neutral, or unpleasant prospective memory cues). The ongoing task stimuli in each session were pictures from all three valences. Each session consisted of five blocks of 55 ongoing stimuli and 5 prospective memory cues (in total 300 stimuli per session). The ongoing task stimuli were presented in a pseudo-randomized way. We created three randomized orders of the ongoing task stimuli, one for each prospective memory session. In each block, 24% of the ongoing task stimuli were one-back hits. Each block started with the encoding phase of prospective memory cues followed by a summary of the task instructions before the respective block started. The three prospective memory sessions were presented in a counterbalanced order between participants to control for possible order effects.

At the end of the experiment, participants worked on a recognition task to assess retrospective memory for the prospective memory cues. Participants were asked to recognize the prospective memory cues. The task consisted of 75 former prospective memory cues (25 cues per session) and 75 ongoing stimuli as distractors. Participants had to press either the key “N” or “M” to indicate if the picture was a former prospective memory cue or an ongoing stimulus. The response-key mapping was counterbalanced across participants. The experiment was run using the software Eprime 2.0 (Psychology Software Tools).

### Recording of the Electrophysiological Data

The electrophysiological data was recorded continuously for the baseline block, the three prospective memory sessions, and the recognition block using the Active-Two BioSemi system with 32 AG/AgCl active scalp electrodes. Electrodes were distributed on the head according to the 10–20 system using head caps with electrode holders (Fp1, Fpz, Fp2, F7, F3, FZ, F4, F8, FC3, FCZ, FC4, T7, C3, CZ, C4, T8, CP3, CPZ, CP4, P7, P3, PZ, P4, P8, PO3, POZ, PO4, O1, OZ, O2, left mastoid, right mastoid). Additionally, we applied six facial electrodes near the outer canthi as well as below and above the pupils to record eye movements (LO1, LO2, IO1, IO2, SO1, SO2). Data were recorded with the ActiView BioSemi software (BioSemi Active-Two, V.O.F., Amsterdam, Netherlands) and digitized at a sampling rate of 2048 Hz in a bandwidth filter of 0–417 Hz. All electrode offsets were held between ±20 μV. Processing of the data was accomplished using the EEGlab 14.1.1 toolbox (Delorme and Makeig, 2004) for Matlab (Version R2016, MathWorks, Natick, MA, United States). The data were downsampled to 512 Hz and low-pass filtered with 30 Hz. Following, the data were further downsampled to 256 Hz and high-pass filtered with 0.1 Hz. In the next step, the data were segmented in epochs of 200 ms before stimulus onset and 2400 ms after stimulus onset. Artifact rejection was performed using independent component analysis (ICA) algorithm from the EEGlab toolbox. Afterward, epochs were re-segmented from 200 ms before stimulus onset until 1400 ms after stimulus onset for stimuli with correct responses. Baseline correction was applied using the 200 ms pre-stimulus interval. Epochs showing activity above of ±75 μV were excluded. All electrodes were re-referenced offline to the average of the two mastoid electrodes. ERPs were averaged for each subject and condition for ongoing task trials, prospective memory cues and hits and recognition trials. We obtained the following numbers of artifact-free trials for the average: prospective memory cues at encoding per session *M* = 24.50, *SD* = 1.82 (younger adults: *M* = 24.19, *SD* = 2.42; older adults: *M* = 24.82, *SD* = 0.63); ongoing task trials per valence *M* = 87.44; *SD* = 11.19 (younger adults: *M* = 84.82, *SD* = 14.83; older adults: *M* = 90.33, *SD* = 2.29); prospective memory hits per session *M* = 20.07, *SD* = 5.11 (younger adults: *M* = 20.46, *SD* = 4.65; older adults: *M* = 19.61, *SD* = 5.60); and recognition trials per task type and valence *M* = 18.75, *SD* = 5.85 (younger adults: *M* = 19.09, *SD* = 5.46; older adults: *M* = 18.33, *SD* = 6.30). We had to exclude two younger adults and one older adult from the electrophysiological analyses due to bad signal.

### Data Analysis

#### Behavioral Data Analysis

The behavioral data were analyzed separately for the prospective memory task and the ongoing task performance. We conducted mixed ANOVAs with age group (two: younger adults and older adults) as between-subjects’ factor and valence of the prospective memory session (three: pleasant, neutral, and unpleasant) and valence of the ongoing task stimuli (three: pleasant, neutral, and unpleasant) as within-subject factors. The ANOVAs were conducted separately for the accuracy rates and the reaction times of prospective memory hits (i.e., correct response after detecting the prospective memory cue) and ongoing task hits (i.e., correct same/different judgment). Greenhouse-Geisser corrections were applied whenever necessary and degrees of freedom were adapted accordingly. Alpha level was set to 0.05. Significant main effects and interactions were explored using *post hoc t*-tests. *Post hoc* tests were corrected for multiple testing using the Bonferroni adjustment: for the subsequent *t*-tests, all *p*-values were multiplied by the number of comparisons made (indicated as *p*_adj_).

#### Electrophysiological Data Analysis

The electrophysiological data were analyzed with partial least square (PLS) analysis (Lobaugh et al., 2001; McIntosh and Lobaugh, 2004). PLS is a multivariate statistical technique that identifies latent differences in ERP amplitudes between experimental conditions across time and space. As input, PLS analyzes the cross-block covariance between a set of orthonormal contrasts reflecting the experimental design of the study and the ERP data, with ERP data consisting of averaged signals per subject and condition in rows and the amplitudes of all time points and electrodes (i.e., 0–1400 ms) in the columns. We used the 30 head electrodes without mastoids for the analyses. PLS was conducted using the PLSGUI for Matlab^1^. The PLS yields a relation between a cohesive pattern of ERP activity over the scalp and a specific experimental effect, which is represented by the optimal contrast across the task conditions, or design scores. In particular, the following three outputs were analyzed and interpreted: (1) task-related variance of latent variables (LVs), (2) ERP saliences, and (3) design scores. The singular value for an LV corresponds to the covariance of the ERP activity with the experimental conditions, expressed in terms of design scores. Each LV explains a progressively lower percentage of the total covariance pattern, until all the covariance is explained. The significance of the LVs values was calculated using a permutation test (1000 replications). An LV was considered as significant at *p* < 0.05. The second and third outputs characterize the structure of the LVs. The design scores describe the contrast between the experimental conditions. Importantly, a greater difference among the distinct design scores reflects a greater difference in the ERP pattern among the relative experimental conditions captured by the corresponding LV. The electrode saliences show where in time (i.e., at which temporal intervals of the epochs) and space (i.e., at which electrodes) the contrast is expressed (Lobaugh et al., 2001; West and Krompinger, 2005). To reduce the bias of possible outliers and to provide a standard error, all data were bootstrapped by randomly resampling participants with replacement 200 times, therefore, allowing to determine the reliability of the saliences identified by the LVs. Bootstrap ratios > 3 were chosen as the cut off for stable non-zero saliences.

We conducted four separate PLS analyses to analyze the ERP effects for the four phases of prospective remembering in younger and older adults:

(1) (1) The first analysis referred to the *encoding phase* and considered ERPs elicited by pleasant, neutral, and unpleasant prospective memory cues that resulted in a later correct response (prospective memory hit). One younger subject had to be excluded because of not enough data points.
(1) (2) The second analysis referred to the *maintenance phase* and considered ERPs elicited by pleasant, neutral, and unpleasant ongoing task hits in the three prospective memory sessions. This analysis comprised nine conditions by crossing the three valence levels of the prospective memory sessions and the three different valence conditions of the ongoing task stimuli.
(1) (3) The third analysis referred to the *retrieval phase* and considered ERPs elicited by prospective memory cues and by ongoing task hits. The valence for prospective memory cues and ongoing task hits was matched comparing the same emotional valence for the different trials (pleasant, neutral, and unpleasant). The analysis included six conditions because ongoing stimuli valence and prospective memory session valence was matched. We had to exclude one older participant due to too few prospective memory hits in some of the conditions.
(1) (4) The fourth analysis referred to the *recognition task* and considered the ERPs elicited by prospective memory trials and ongoing task trials in the recognition task. The analysis considered six conditions by crossing the two task types (prospective memory cues and ongoing task stimuli) with the three valence levels (pleasant, neutral, and unpleasant). We had to exclude one younger and three older adults because of missing electrophysiological data.

## Results

### Behavioral Results

**Table 1** depicts the descriptive measures for the behavioral performance rates in younger and older adults.

**Table 1:** Behavioral performance rates for younger and older adults.

	Younger adults			Older adults		
	Valence of the pictures			Valence of the pictures		
	Unpleasant	Neutral	Pleasant	Unpleasant	Neutral	Pleasant
***OT***						
Accuracy *(SD)*						
PM session						
Unpleasant	0.94 (0.13)	0.92 (0.15)	0.91 (0.15)	0.93 (0.11)	0.93 (0.09)	0.92 (0.08)
Neutral	0.93 (0.08)	0.93 (0.09)	0.93 (0.12)	0.96 (0.02)	0.96 (0.03)	0.98 (0.03)
Pleasant	0.96 (0.03)	0.95 (0.04)	0.96 (0.04)	0.94 (0.05)	0.96 (0.05)	0.96 (0.05)
Reaction time *(SD)*						
PM session						
Unpleasant	769 (184)	725 (159)	743 (165)	932 (184)	846 (144)	872 (163)
Neutral	747 (166)	735 (169)	734 (163)	879 (118)	880 (118)	851 (114)
Pleasant	760 (164)	725 (164)	777 (185)	868 (120)	828 (118)	898 (130)
***PM cues***						
Accuracy *(SD)*	0.87 (0.15)	0.86 (0.17)	0.83 (0.18)	0.76 (0.30)	0.86 (0.22)	0.82 (0.17)
Reaction time *(SD)*	442 (109)	452 (112)	454 (104)	472 (150)	505 (117)	520 (142)
**Recognition task**						
Accuracy *(SD)*						
PM trials	0.74 (0.21)	0.70 (0.24)	0.69 (0.25)	0.64 (0.28)	0.72 (0.24)	0.64 (0.24)
OT trials	0.88 (0.20)	0.92 (0.18)	0.90 (0.17)	0.88 (0.11)	0.90 (0.09)	0.92 (0.13)
Reaction time *(SD)*						
PM trials	942 (129)	949 (147)	982 (158)	1077 (182)	991 (138)	992 (268)
OT trials	856 (121)	845 (106)	851 (124)	912 (95)	878 (101)	945 (128)


*OT, ongoing task; PM, prospective memory task; SD, standard deviation; Reaction time in ms.*

#### Prospective Memory Performance

First, we conducted an ANOVA on accuracy rates for the prospective memory hits including the factors age group (two: younger adults and older adults) and prospective memory session (three: pleasant, neutral, and unpleasant). The ANOVA did not reveal significant main effects for age group [*F*(1,42) = 0.491; *p* = 0.487] or prospective memory session [*F*(1.71,71.93) = 1.525; *p* = 0.226]. There were no performance differences between younger and older adults as well as between the three different valence conditions of the prospective memory cues. The interaction did not reach significance [*F*(1.71,71.93) = 2.783; *p* = 0.077] but showed descriptively that younger and older adults differed in their performance for negative prospective memory cues with younger adults performing better than older adults.

In a second step, we analyzed the reaction times for prospective memory hits. Although younger adults performed descriptively faster than older adults, the ANOVA revealed no significant main effect for age group [*F*(1,42) = 2.587; *p* = 0.115]. Also neither the main effect of prospective memory valence condition [*F*(2,84) = 1.657; *p* = 0.197] nor the interaction reached significance [*F*(2,84) = 0.567; *p* = 0.569].

#### Ongoing Task Performance

In a first step, we analyzed correct ongoing task performance including the factors age group (two: younger adults and older adults), valence of the prospective memory session (three: pleasant, neutral, and unpleasant), and valence of the ongoing task stimuli (three: pleasant, neutral, and unpleasant). The ANOVA revealed a significant interaction of the valence of the ongoing task stimuli and age group [*F*(2,84) = 3.959; *p* = 0.023; $\eta_{p}^{2}$ = 0.086] and a significant interaction of prospective memory session and valence of the ongoing task stimuli [*F*(3.24,136.00) = 6.004; *p* = 0.001; $\eta_{p}^{2}$ = 0.125]. The main effect of prospective memory session approached significance [*F*(1.51,63.32) = 3.466; *p* = 0.050; $\eta_{p}^{2}$ = 0.076]. The main effects of age group and valence of the ongoing task stimuli as well as the interaction of prospective memory session and the three-way interaction did not reach significance (all *p* > 0.246).

*Post hoc* comparisons for the interaction of age group by valence of the ongoing task stimuli did not held significant at the follow-up analyses (*p*_adj_ > 0.999). For the interaction of prospective memory session by ongoing task valence, the *post hoc* comparisons showed that performance for pleasant ongoing task trials was slightly higher compared to neutral ongoing task trials (*p*_adj_ = 0.025) in the neutral prospective memory session (for all other comparisons *p*_adj_ > 0.096). Performance of pleasant ongoing task trials was better than for unpleasant ongoing task trials in the pleasant prospective memory session (*p*_adj_ = 0.038; for all other comparisons *p*_adj_ > 0.674). However, there were no differences in the unpleasant prospective memory session between the valence levels of the ongoing task trials (all *p*_adj_ > 0.068).

In a second step, we analyzed the ANOVA of the reaction times for correct ongoing task trials including the factors age group, valence of the prospective memory session, and valence of the ongoing task stimuli. The ANOVA revealed significant main effects of age group [*F*(1,42) = 9.416; *p* = 0.004; $\eta_{p}^{2}$ = 0.183] and valence of the ongoing task stimuli [*F*(2,84) = 33.441; *p* < 0.001; $\eta_{p}^{2}$ = 0.443] as well as significant interaction of valence of the prospective memory session by valence of the ongoing task stimuli [*F*(4,168) = 29.548; *p* < 0.001; $\eta_{p}^{2}$ = 0.413] and a significant three-way interaction of all three factors [*F*(4,168) = 4.212; *p* = 0.003; $\eta_{p}^{2}$ = 0.091]. All other main effects and interactions did not turn significant (all *p* > 0.309).

Older adults performed slower than younger adults (*p*_adj_ = 0.004) and participants performed overall faster for neutral ongoing task trials, intermediate for pleasant ongoing task trials, and slowest for unpleasant ongoing task trials (all *p*_adj_ < 0.003). To break down the results of the interactions, we will focus on the three-way interaction by separating the two age groups.

In the unpleasant prospective memory session, younger and older adults showed a similar reaction time pattern. Younger adults were fastest for neutral ongoing task trials, intermediate for pleasant ongoing task trials, and slowest for unpleasant ongoing task trials (neutral–pleasant: *p*_adj_ = 0.050; neutral–unpleasant: *p*_adj_ = 0.001; and pleasant–unpleasant: *p*_adj_ = 0.026). Older adults were fastest for the neutral ongoing task trials but reaction times did not differ from pleasant ongoing task trials, and they performed the slowest for the unpleasant ongoing task trials (neutral–pleasant: *p*_adj_ = 0.177; neutral–unpleasant: *p*_adj_ = 0.002; and pleasant–unpleasant: *p*_adj_ < 0.001).

In the neutral prospective memory session, younger adults did not show reaction time differences between the three valence levels of the ongoing task trials (all *p*_adj_ > 0.255). Older adults performed faster for pleasant ongoing task trials than unpleasant trials (*p*_adj_ = 0.013), but there were no differences toward the neutral ongoing task trials (all *p*_adj_ > 0.065).

Finally, in the pleasant prospective memory session, both age groups performed faster for neutral ongoing task trials compared to the emotional trials (all *p*_adj_ < 0.001). Additionally, older adults performed faster on unpleasant trials compared to pleasant trials (*p*_adj_ = 0.001), whereas younger adults did not show that difference (*p*_adj_ = 0.108).

#### Recognition Task

For the recognition task, we conducted a mixed ANOVA including the factors age group (two: younger adults and older adults), task type (two: prospective memory cues and ongoing task trials), and valence of the stimuli (three: pleasant, neutral, and unpleasant). The ANOVA for the accuracy rates showed only a significant main effect of task type [*F*(1,42) = 33.239; *p* < 0.001; $\eta_{p}^{2}$ = 0.442] but no other effect or interaction turned significant (all *p* > 0.134). Performance rates were better for the ongoing task trials than the prospective memory trials.

We conducted a second ANOVA with the same factors for the reaction times. The ANOVA resulted in a significant main effect of age group [*F*(1,41) = 4.376; *p* = 0.043; $\eta_{p}^{2}$ = 0.096] and a significant main effect of task type [*F*(1,41) = 38.833; *p* < 0.001; $\eta_{p}^{2}$ = 0.486]; however, no other effect turned significant (all *p* > 0.133). Younger adults performed faster than older adults. Performance rates were faster for ongoing task trials than prospective memory trials.

### Electrophysiological Results

#### Encoding Phase

**FIGURE 1:**
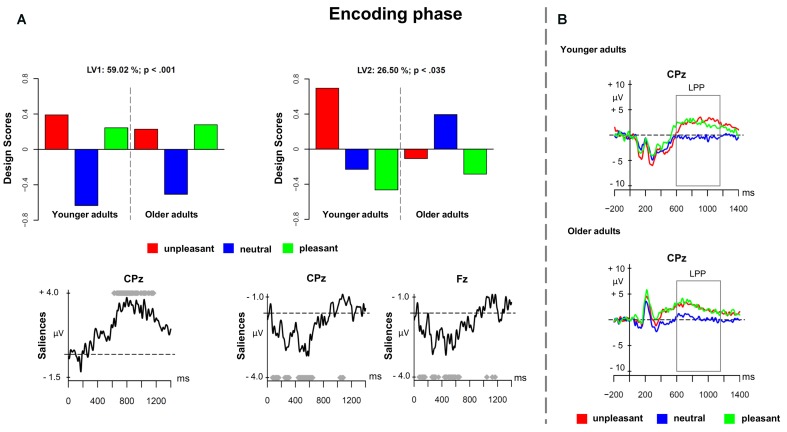
Partial least square (PLS) results and grand averages for the encoding phase. **(A)** Design scores and event-related potential (ERP) saliences for the comparison of the three valence conditions of the prospective memory cues separately for younger and older adults. **(B)** Grand-averaged ERPs for younger and older adults at electrode CPz.

The first PLS analysis (*encoding phase*) involved ERPs elicited by unpleasant, neutral, and pleasant pictures in the encoding phase. **Figure 1** depicts the results of the PLS analysis for the encoding phase. The analysis showed two significant LVs (*p* < 0.001 and *p* < 0.035), which, respectively, accounted for 59.02 and 26.50% of the cross-block covariance. The LV1 captured a contrast between emotional pictures (both unpleasant and pleasant) and neutral pictures, in both younger and older adults (**Figure 1A** depicts the design scores and ERP saliences). The finding replicated the results from Cona et al. (2015a) and extended them to older adults. The LV1 reflected a sustained positivity associated with emotional stimuli over parietal and centro-parietal electrodes in the time window roughly between 600 and 1200 ms after stimulus onset representing the LPP (**Figure 1B** depicts the ERPs for younger and older adults at electrode CPz). Younger and older adults seemed to recruit more attentional resources for pleasant and unpleasant cues compared to neutral cues expressed in the increased LPP component for emotional material.

The LV2 showed age-related differences in ERPs for the encoding of emotional versus neutral prospective memory cues. **Figure 1A** depicts the design scores and displays age-related differences. In younger adults, the unpleasant prospective memory cues differed substantially compared to both neutral and pleasant cues, suggesting the presence of a negative bias. However, in older adults, there was no such difference present. Regarding the spatial distribution, the modulations associated with the LV2 were widespread over the scalp, involving both earlier and later ERP components. More precisely, emotional stimuli led to enhanced amplitudes in the negative components that occurred in the time windows between 100–180 ms (i.e., N1), 250–350 ms (i.e., N2), and 420–600 ms (i.e., N3). The negative bias – that was expressed only in younger adults – was visible as increased negativity for unpleasant prospective memory cues in the three negative components. This negative modulation could reflect the early posterior negativity (EPN), which is associated with visual processing of emotional stimuli.

#### Maintenance Phase

**FIGURE 2:**
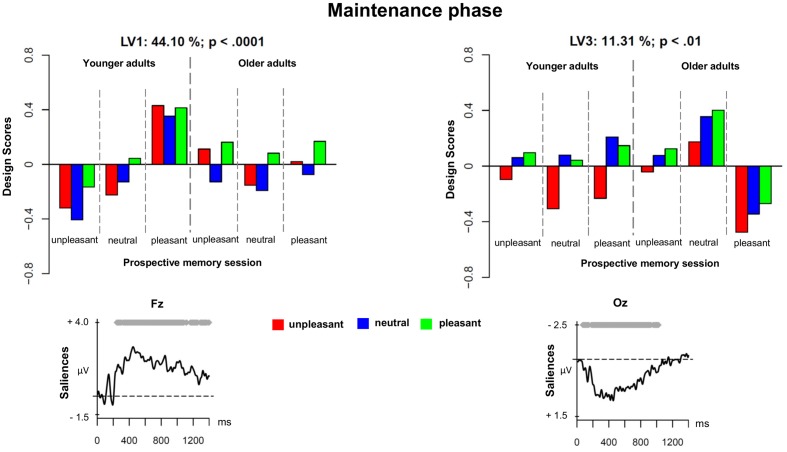
Design scores and ERP saliences for the comparison of the three prospective memory sessions and three levels of ongoing task trials during the maintenance phase separately for younger and older adults.

**FIGURE 3:**
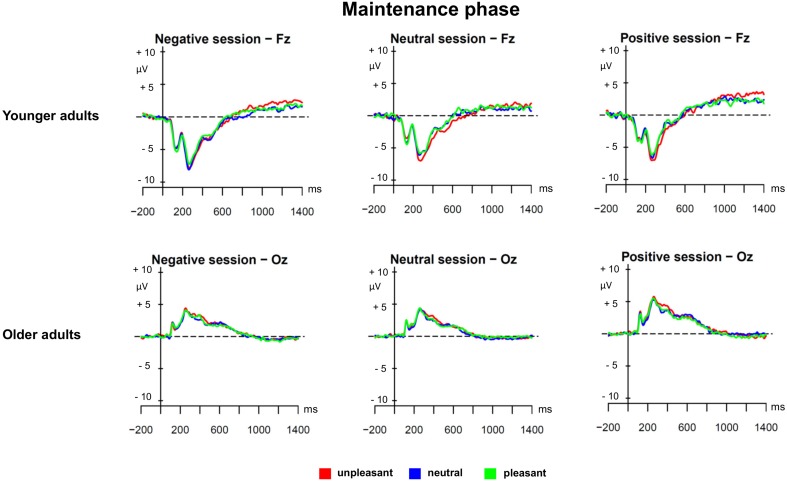
Grand averages for the maintenance phase separately for younger and older adults and the three prospective memory sessions.

The second PLS analysis (*maintenance phase*) was applied to the ERPs evoked by unpleasant, neutral, and pleasant ongoing task stimuli in the three prospective memory sessions (unpleasant, neutral, and pleasant) in younger and older adults. **Figure 2** shows the results of the PLS analysis for the maintenance phase, and **Figure 3** depicts the ERPs for both age groups and the three different prospective memory sessions.

Three LVs were found to be significant (all *p*s < 0.01). The LV1 accounted for 44.10% of the cross-block covariance and reflected, in younger adults, a difference between monitoring for the presence of unpleasant versus pleasant prospective memory cues. Regardless of the valence of the ongoing stimuli, monitoring for pleasant prospective memory cues led to an increased sustained positivity over frontal and fronto-central regions roughly between 400 and 1000 ms. This frontal and fronto-central slow wave could represent two overlapping components: long-lasting sustained activity associated with strategic monitoring and the LPP. The long-lasting activity that seemed to be associated with strategic monitoring processes might indeed reflect a retrieval mode (Guynn, 2003), which is a process necessary to actively maintain the intention in memory (West, 2011; Cona et al., 2012a,b; Czernochowski et al., 2012). The LPP component could reflect top-down processing and the allocation of attentional resources toward the emotional material, specifically, pleasant material (cf. Hajcak et al., 2006).

The pattern observed for older adults was less clear. The ERP modulations associated with maintaining the pleasant prospective memory cues in older adults were captured by the LV3. For this reason, we decided to present LV3 first before describing the LV2.

As displayed by **Figure 2**, the LV3 accounted for 11.32% of the cross-block covariance and captured, in older adults, a contrast between the pleasant prospective memory session and the unpleasant prospective memory session, independently of the valence of the ongoing task stimuli. When comparing the ERP salience maps captured by LV1 and LV3, it was evident that there was a differential effect for maintaining pleasant prospective memory cues in younger compared to older adults. In younger adults, the ERP correlates of this process were expressed in late windows in a small region over frontal and fronto-central sites that likely represented the LPP. In older adults, the maintenance of pleasant prospective memory cues was associated with a very widespread sustained modulation over posterior and occipital sites that started from 150 ms and lasted until 1000 ms (see **Figure 3** for the ERPs in younger and older adults separately). This slow-wave activity in older adults suggests a positivity bias for the maintenance of pleasant prospective memory cues in older adults, which occured already in the early stages of processing as it is evidenced already in the earlier components.

Interestingly, in younger adults, the LV3 captured ERPs that were associated with unpleasant ongoing task stimuli, regardless of the prospective memory session. This corroborates the results evidenced in the analysis of the encoding phase, highlighting a negative bias selectively for younger adults.

We decided to not describe and discuss the LV2 in detail given that the pattern of the design scores is less clear and LV2 is only expressed in a late time window (after 1200 ms). However, with caution, this LV could reflect the presence of a negative bias for younger adults.

#### Retrieval Phase

**FIGURE 4:**
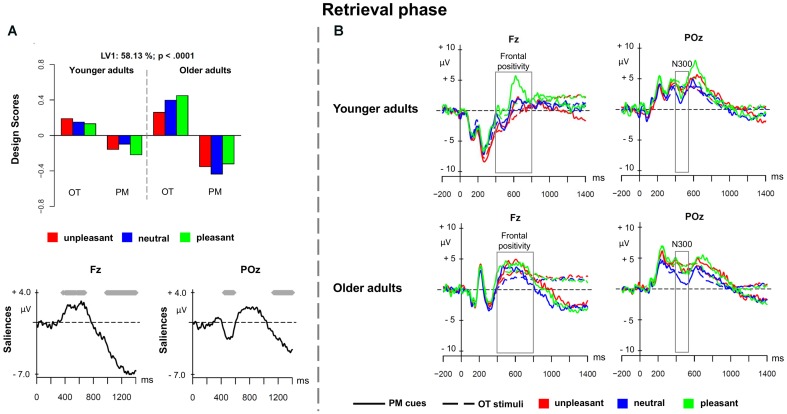
Partial least square results and grand averages for the retrieval phase. **(A)** Design scores and ERP saliences for the comparison of the three valence conditions of the prospective memory cues and the corresponding ongoing task trials separately for younger and older adults. **(B)** Grand-averaged ERPs for younger and older adults at electrodes Fz and POz comparing prospective memory cues with ongoing task stimuli at the three valence conditions. PM, prospective memory; OT, ongoing task.

The third PLS analysis (*retrieval phase*) was performed on the ERPs elicited by the prospective memory cues and by the ongoing task stimuli with the same emotional valence of the PM cues in a given session, in younger and older adults. **Figure 4A** depicts the PLS results for the retrieval phase and **Figure 4B** shows the associated ERPs in younger and older adults. This analysis revealed a significant LV1 (*p* < 0.0001) that accounted for 58.13% of the cross-block covariance. LV1 reflected a contrast between prospective memory cues and ongoing task stimuli, revealing that such difference was more expressed in older than younger adults, and especially for pleasant pictures.

The LV1 captured an increased positivity for prospective memory cues, particularly pronounced over frontal regions, in the time window between 400 and 800 ms suggesting a frontal positivity, and a slow-wave negativity at 1000 ms, widely expressed over the scalp. Over parieto-occipital and occipital sites, a transient negativity occurred between 430 and 550 ms that characterized the prospective memory cues and may reflect the N300. These components were more expressed in older than younger adults. The N300 and the frontal positivity are correlates of prospective memory cue detection. The results suggest that older adults showed a positivity bias to detect the pleasant prospective memory cues (N300) and to switch from the ongoing task to the intention (frontal positivity). The late negative slow-wave activity suggests the presence of the parietal positivity and could be associated with task set coordination between the intention execution and the ongoing task.

#### Recognition Task

**FIGURE 5:**
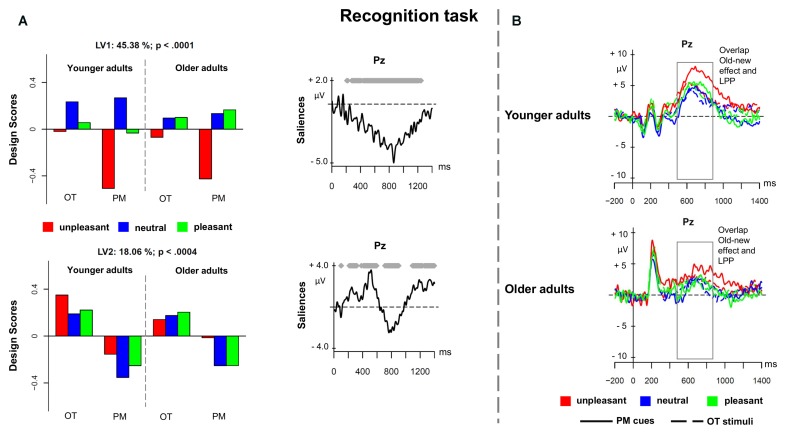
Partial least square results and grand averages for the recognition task. **(A)** Design scores and ERP saliences for the comparison of the three valence conditions of the prospective memory cues and the ongoing task trials separately for younger and older adults. **(B)** Grand-averaged ERPs for younger and older adults at electrode Pz comparing prospective memory cues with ongoing task stimuli at the three valence conditions. PM, prospective memory; OT, ongoing task.

The fourth PLS analysis (*recognition task*) included the ERPs elicited by prospective memory cues and ongoing task stimuli in the recognition task, for younger and older adults. **Figure 5** shows the results for the PLS analysis on the recognition task. The analysis showed two significant LVs (*p* < 0.001 and *p* < 0.004) that accounted for 45.38 and 18.06% of the cross-block covariance, respectively. As similarly found in the study by Cona et al. (2015a), LV1 distinguished the unpleasant recognized PM cues from the other conditions (**Figure 5A**). The difference in the magnitude of the design scores suggests that such difference was mainly expressed in younger than older adults. The modulation represented an increased sustained positivity for the unpleasant prospective memory cues in the time window between 500 and 900 ms suggesting a negativity bias in memorizing unpleasant material. This component was widely expressed over the scalp, and might likely be the result of the overlap between the recognition old–new effect and the LPP (**Figure 5B**).

LV2 distinguished the recognized prospective memory cues from the recognized ongoing task stimuli in both age groups (**Figure 5A**). LV2 captured a latency difference in the slow-wave positivity occurring between 500 and 1000 ms over frontal and parietal regions, which might be the result of distinct overlapping components, such as the P3b that might reflect a higher amount of attentional resources allocated for the prospective memory cues, and the parietal old–new effect, that is mainly associated with strategic recognition processes (West and Wymbs, 2004; West, 2011). Prospective memory cues seemed indeed to elicit a delayed sustained positivity compared to the ongoing stimuli, in line with the behavioral results. The differences in the encoding of the two stimuli groups might account for that difference and the sustained activity for the prospective memory stimuli.

## Discussion

The aim of the present study was to investigate the influence of emotional material on prospective memory in younger and older adults. We used an established prospective memory task (Cona et al., 2015a) that manipulated the emotional valence of the prospective memory cues and the ongoing task. Participants had to encode either pleasant, neutral, or unpleasant prospective memory cues before they worked on a picture one-back ongoing task with pleasant, neutral, and unpleasant pictures. Previous studies indicated that older adults might benefit from emotional cues reducing or even eliminating age differences (e.g., Altgassen et al., 2010; Schnitzspahn et al., 2012). Here, it had been suggested that emotional prospective memory cues serve as highly distinct or salient cues which lead to spontaneous retrieval. Spontaneous retrieval is less resource demanding and usually intact in older adults. Alternatively, emotional cues could increase the importance of detecting the cues, and thus, enhance more strategic monitoring, that is resource demanding and decreased in older adults. To follow up on this debate, we assessed not only behavioral performance but also included electrophysiological measures to uncover the underlying mechanism.

### Behavioral Results

Contrary to our assumption, the behavioral analyses showed no age differences for the prospective memory performance. Younger and older adults performed at similar rates when they had to respond to the prospective memory cues. Furthermore, there were also no performance differences between the three valence conditions. The lack of an age difference was somewhat surprising as it is well established in the literature that prospective memory declines with increasing age (e.g., Ihle et al., 2013). We had assumed that age differences should be small or even eliminated for the emotional conditions, but it was also the case for the neutral condition that resembles a standard prospective memory task. Similar to our results, Cona et al. (2015a) did not find differences between the three valence conditions on prospective memory accuracy in younger adults. They argued that the task was not too demanding for younger adults. Indeed, the task is a so-called focal prospective memory task. Focality is a task property that stimulates either spontaneous retrieval or strategic monitoring depending on the specification (McDaniel and Einstein, 2000). A task is considered as focal, when the detection of prospective memory cue and the ongoing task share the same processes; thus, the retrieval should occur rather spontaneous as the prospective memory cue is processed directly along with the ongoing task. In the present task, participants had to work on a picture one-back task, so they had to process the pictures that also served as prospective memory cues. It is assumed that age differences are reduced under focal conditions (Kliegel et al., 2008b; Ihle et al., 2013).

The findings from the ongoing task revealed more about the influence of the emotional material on performance. Again, we did not find age differences for the ongoing task accuracy. The task itself was not too demanding. It was desirable to use an ongoing task that is equally demanding for both age groups to better compare the underlying mechanisms. For both age groups, performance for pleasant trials was higher than for neutral and unpleasant trials in the neutral and pleasant prospective memory session. The finding indicates a positivity bias that is usually found in older adults. The positivity bias describes the finding that older adults show a processing bias toward positive information (e.g., Mather and Carstensen, 2005). The reaction time data also support this conclusion. In general, older adults showed a slowing in their response times compared to younger adults, which is a widely confirmed finding (e.g., Salthouse, 1996). However, older adults performed faster for pleasant trials than unpleasant or neutral trials in the condition with neutral prospective memory cues, whereas younger adults did not differ between the three valence conditions of the ongoing task. Similar to the results from Cona et al. (2015a), the younger and older adults showed an SSIE (Cohen et al., 2012). Both age groups showed the highest reaction times for unpleasant ongoing task trials when they had to keep in mind unpleasant prospective memory cues. The SSIE indicates that both age groups increased attentional resources for target checking that is a monitoring process where incoming stimuli were evaluated of being a prospective memory cue or not (Guynn, 2003). Target checking is an indication for strategic monitoring. Additionally, older adults showed an SSIE effect for pleasant cues and ongoing trials as well, whereas the effect did not occur in younger adults indicating again toward a positivity bias in older adults. In contrast to Cona et al. (2015a), we could not follow up on the findings regarding the recognition task. We only found an advantage for the ongoing task stimuli.

In conclusion, the behavioral findings do not yet allow answering our research question regarding the modulating role of emotional material on prospective remembering. They are partly in line with the beneficial effects reported by Altgassen et al. (2010), who did also not find age differences between younger and older adults in a prospective memory task with emotional cues.

Besides the behavioral measures, we recorded the ERPs to receive a better understanding of the neural modulations that might occur due to the influence of emotional material. The ERP analyses were especially helpful to explore the underlying mechanisms of the different phases of prospective remembering and allowed for differentiated investigation of encoding, maintaining, and retrieving intentions.

### Electrophysiological Results

The first phase of prospective remembering refers to the encoding of the prospective memory cue. Encoding *per se* is usually not assessed as behavioral response; therefore, the ERP results offer important insights. We found evidence for an LPP that was specific to pleasant and unpleasant prospective memory cues but not for neutral cues. The LPP is associated with sustained attention for emotional material indicating that both age groups showed comparable encoding effort for the pleasant and unpleasant cues. Moreover, younger and older adults showed enhanced encoding for the emotional prospective memory cues suggesting that emotional material is processed as a priority not only in younger adults (Dolcos and Cabeza, 2002), but also in older adults.

Interestingly, there was also evidence for age-related differences during encoding. Younger adults showed increased negative activity for unpleasant cues (but not pleasant or neutral cues) in three different time windows widespread over the scalp, whereas older adults did not show differences between the three valence conditions. It could reflect the EPN that is associated with visual processing of stimuli (e.g., Schupp et al., 2003a,b, 2004). The EPN is also sensitive to the arousal of the stimulus material (Olofsson et al., 2008), which might indicate that this negative bias in the younger adults might be linked to the arousing nature of the unpleasant stimuli. This negativity bias in younger adults might reflect an evolutionary bias toward threating stimuli (Öhman and Mineka, 2001).

Regarding the next phase, the maintenance phase, the results showed two interesting findings. In younger adults, we found evidence for increased monitoring for pleasant prospective memory cues independent of the valence of the ongoing task indicated by a frontal-central distributed sustained positivity. Similarly, West (2011) and Cona et al. (2012b) reported sustained positive activity when participants have to hold in mind an intention indicating a retrieval mode (Guynn, 2003). The retrieval mode describes a general state of readiness that the prospective memory cue will arrive. In older adults, the sustained activation associated with monitoring for pleasant cues occurred over posterior and occipital sites. However, it is to note that the EEG has only a poor spatial resolution and we included only 32 electrodes, and therefore, conclusions on the spatial origins of the effects have to be considered with caution. Nevertheless, the finding is in line with our behavioral result, where older adults showed an SSIE for pleasant prospective memory cues and ongoing task trials indicating monitoring for pleasant cues in older adults. Furthermore, we also found an LV specific for unpleasant ongoing trials in younger adults that could reflect the LPP indicating again the negativity bias in younger adults with enhanced monitoring for the unpleasant prospective memory cues.

Interestingly, the findings for the maintenance phase indicate that emotionally valenced cues augment strategic monitoring in both younger and older adults. Contrary to the conclusions by Altgassen et al. (2010), our findings indicate that the detection of emotional cues does not only rely on spontaneous processing but also on more strategic monitoring for the emotional prospective memory cues, which is supported by the allocation of attentional resources toward the emotional cues (Olofsson et al., 2008). The present findings are in line with the theoretical account of cognitive control in aging that postulates two types of cognitive control: proactive control and reactive control (Braver et al., 2001; Braver and West, 2008; Braver, 2012). Intentional behavior can either be driven top-down by proactive control that is a state of anticipation that cognitive control is necessary similar to strategic monitoring; or goal-directed behavior can be driven bottom-up by reactive control that is the activation of cognitive control whenever it is necessary or triggered by external stimuli similar to spontaneous processing. The specificity toward pleasant prospective memory cues might indicate some selective proactive control processes that might be triggered top-down by motivational influences (Braver, 2012).

Regarding the retrieval phase, we found prospective memory specific ERPs that differed from the ongoing task trials. More interestingly, the ERPs were more expressed in older adults, and especially for pleasant prospective memory cues. There was transient negativity in the time window of 430–550 ms, which reflects the N300 and is associated to cue detection. There was also an increased positivity over frontal regions that might reflect the frontal positivity. Finally, there was a later negative slow wave over parieto-occipital sites that might belong to the parietal positivity complex and seems to be related to the coordination between the ongoing task and the prospective memory task (Bisiacchi et al., 2009). Interestingly, these effects were more expressed in the older adults’ group than in the younger adults. Previous research on non-emotional material found rather attenuated activity in older adults compared to younger adults (West et al., 2002). Importantly, the effects were mainly captured for pleasant cues indicating the positivity bias in older adults that is widely found for the influence of emotional material on attention and memory (Reed and Carstensen, 2012; Reed et al., 2014). It suggests that at the retrieval phase, there seems to be rather automatic capture of attention toward the pleasant cues that facilitates cue detection and retrieval. The elevated activity in the older adults might be an expression of reactive control in this age group triggered by the pleasant (positively biased) prospective memory cues indicating spontaneous retrieval processes (Altgassen et al., 2010). The advantage for pleasant cues compared to neutral and unpleasant cues in the older adults’ group follows up on the finding that neural reactivity toward negative material decreases with increasing age during adulthood (Kisley et al., 2007). The authors investigated the shift from the negativity bias toward the positivity bias in the adulthood lifespan and could show that the negativity bias decreases with increasing age in neural responsivity. The authors found a negative correlation between age and the amplitude of the LPP for unpleasant material but not for neutral and pleasant material. The authors explained their finding with the socioemotional selectivity theory and the proposed shift toward positive information in old adulthood.

Finally, the ERP analysis for the recognition task revealed a negativity bias for the unpleasant prospective memory cues in the younger adults. Although we did not obtain a significant effect, descriptively the behavioral results for the accuracy rates in prospective memory cues tended in the same direction similarly to the results obtained by Cona et al. (2015a). More precisely, this ERP might reflect the recognition old-new effect for prospective memory cues overlapping with the LPP for unpleasant material. Interestingly, the behaviorally delayed response rates for the prospective memory cues were also captured by a delayed sustained positivity.

### Limitations and Outlook

There are some limitations to consider when interpreting the present results. We did not obtain an age effect for the prospective memory performance or the ongoing task performance, which contradicts the literature on age effects in prospective memory. One reason, that was already discussed earlier, might have been the rather easy one-back task as ongoing activity. However, it allowed for having comparable performance rates to investigate age specific effects on the ERP activity. The earlier described decreased activity that is often observed in older adults might also result from increased task demands in the other studies. The performance rates for the prospective memory task, however, suggest that ceiling effects did not drive our results.

The observed spatial differences in ERPs between younger and older adults need further exploration. The EEG is not the appropriate method to investigate spatial sources for the effects although there exists a useful algorithm for source localization (for a review, see Michel et al., 2004). Future studies should also address the role of strategic monitoring during the intention maintenance phase. So far, prospective memory research focused mainly on the retrieval or the encoding phase, mainly because behavioral measures can only be collected for overt behavior. However, using electrophysiological measures, it would be possible to map the neural underpinnings of strategic monitoring during a prospective memory task. Our findings and those by others (West et al., 2011; Cona et al., 2012b) offer first indications for a retrieval mode during the maintenance phase and increased monitoring (e.g., expressed by the sustained positive activity).

## Conclusion

Taken together, the present results suggested that prospective memory performance and the underlying neural processing can be modulated by emotional material. Furthermore, by looking at the entire process of prospective remembering including encoding, maintaining, and retrieving of intentions, we could identify differential mechanisms in younger and older adults. More importantly, our results have conceptual implications for the understanding of emotional influences on prospective memory. Our results showed that emotional material fosters strategic monitoring during the maintenance phase and spontaneous processing during the retrieval phase in both age groups. Contradictory to our assumption, monitoring activity was not reduced in older adults. More importantly, the emotional cues seemed to show enhanced processing and a benefit in attention allocation toward the emotional cues. The present findings suggest that emotional cues indeed boost the relevance of the cues, and thus foster attentional monitoring. On the other side, the increased activity during retrieval in older adults supports the notion that emotionally valenced cues increase their distinctiveness, and thus, support the rather spontaneous detection and retrieval (McDaniel and Einstein, 2000; Altgassen et al., 2010). These conclusions are in line with the attention-to-delayed-intention model by Cona et al. (2015b) that provides a theoretical account for the neuroanatomical foundation of prospective remembering including the prefrontal cortex and the mediotemporal lobe and the integration of strategic monitoring and spontaneous retrieval.

Furthermore, our results showed age-related differences in the neural reactivity toward pleasant and unpleasant prospective memory cues. Our findings are in line with the theoretical account of a shift from a negativity bias in younger adults toward a positivity bias in older adults (Carstensen, 2006; Carstensen and DeLiema, 2018). Although our study only investigated cross-sectional data by comparing younger and older adults, the electrophysiological findings suggested a bias in younger adults toward the unpleasant pictures and a preference for the pleasant images in older adults.

In sum, our findings suggest evidence for both assumptions on how emotional material might influence prospective remembering in older adults and even overcoming age differences. However, the role of top-down and bottom-up processing differs with respect to the specific phase of prospective remembering and the emotional valence of the prospective memory cues.

## Ethics Statement

This study was carried out in accordance with the recommendations of ethical guidelines of the Ethics Committee of the Faculty of Psychology and Educational Sciences of the University of Geneva. All subjects gave written informed consent in accordance with the Declaration of Helsinki. The protocol was approved by the Ethics Committee of the Faculty of Psychology and Educational Sciences of the University of Geneva.

## Author Contributions

AH, MK, PB, and GC designed the study. AH and MK collected the data. AH analyzed the behavioral data. GC analyzed the electrophysiological data with the help of AH. AH and GC wrote the paper with edits from MK and PB.

## Conflict of Interest Statement

The authors declare that the research was conducted in the absence of any commercial or financial relationships that could be construed as a potential conflict of interest.

## Footnotes

1 https://www.rotman-baycrest.on.ca
